# The role of CD8+ T cells in endometriosis: a systematic review

**DOI:** 10.3389/fimmu.2023.1225639

**Published:** 2023-07-11

**Authors:** Ana Kisovar, Christian M. Becker, Ingrid Granne, Jennifer H. Southcombe

**Affiliations:** Nuffield Department of Women’s and Reproductive Health, University of Oxford, Oxford, United Kingdom

**Keywords:** CD8, T cell, endometriosis, endometrium, peritoneal fluid

## Abstract

**Background:**

Endometriosis is a chronic disease affecting 6–10% of women of reproductive age. It is an important cause of infertility and chronic pelvic pain with poorly understood aetiology. CD8+ T (CD8 T) cells were shown to be linked to infertility and chronic pain and play a significant role in lesion clearance in other pathologies, yet their function in endometriosis is unknown. We systematically evaluated the literature on the CD8 T in peripheral blood and endometriosis-associated tissues to determine the current understanding of their pathophysiological and clinical relevance in the disease and associated conditions (e.g. infertility and pelvic pain).

**Methods:**

Four databases were searched (MEDLINE, EMBASE, Web of Science, CINAHL), from database inception until September 2022, for papers written in the English language with database-specific relevant terms/free-text terms from two categories: CD8 T cells and endometriosis. We included peer-reviewed papers investigating CD8 T cells in peripheral blood and endometriosis-associated tissues of patients with surgically confirmed endometriosis between menarche and menopause, and animal models with oestrous cycles. Studies enrolling participants with other gynaecological pathologies (except uterine fibroids and tubal factor infertility used as controls), cancer, immune diseases, or taking immune or hormonal therapy were excluded.

**Results:**

28 published case-control studies and gene set analyses investigating CD8 T cells in endometriosis were included. Data consistently indicate that CD8 T cells are enriched in endometriotic lesions in comparison to eutopic endometrium, with no differences in peripheral blood CD8 T populations between patients and healthy controls. Evidence on CD8 T cells in peritoneal fluid and eutopic endometrium is conflicting. CD8 T cell cytotoxicity was increased in the menstrual effluent of patients, and genomic analyses have shown a clear trend of enriched CD8 T effector memory cells in the eutopic endometrium of patients.

**Conclusion:**

Literature on CD8 T cells in endometriosis-associated tissues is inconsistent. Increased CD8 T levels are found in endometriotic lesions, however, their activation potential is understudied in all relevant tissues. Future research should focus on identifying clinically relevant phenotypes to support the development of non-invasive diagnostic and treatment strategies.

**Systematic Review Registration:**

PROSPERO identifier CRD42021233304

## 1 Introduction

Endometriosis is a common gynaecological disease defined as the presence of endometrium-like tissue outside the uterus (1, 2). It affects one in ten females of reproductive age, translating to approximately 190 million worldwide (3). Although the exact pathophysiology of the disease is still unclear, the oldest and most widely accepted explanation is the retrograde menstruation theory proposing that endometrial cells travel through the uterine tubes into the abdominal cavity, adhere to the peritoneum and invade the mesothelium and deeper layers (4). Retrograde menstruation is a physiological process occurring in most women, but only in some do these cells persist and cause inflammation, with potential pelvic pain and infertility. Therefore, additional factors have been suggested to play a role, including altered systemic and local immunity, resulting in a disruption in the removal of endometrial cells from the abdominal cavity (1).

**Figure 1:**
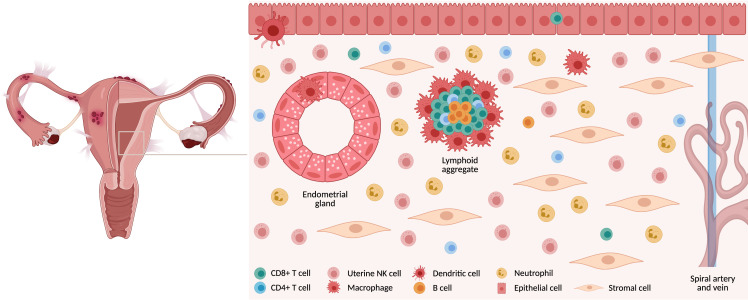
Immune cells in endometrial mucosa. The endometrium is comprised of a variety of cell types. Luminal and glandular epithelium are surrounded by stromal cells, which are interspersed with a variety of immune cells. These are either found as single cells or in lymphoid aggregates. Created with BioRender.com.

Endometrial mucosa is populated by various immune cells (**Figure 1**) which not only provide immunity against pathogens but also help facilitate embryo implantation and pregnancy (5). T cells are the most abundant leukocytes comprising 40–60% in the proliferative phase, decreasing to <10% in the late secretory phase due to the accumulation of natural killer (NK) cells post ovulation (5, 6). Of all T cell populations, approximately 60% are CD8+ T (CD8 T) cells and are found scattered throughout the endometrium either as single cells surrounded by stromal cells, as intraepithelial cells adjacent to the luminal or glandular epithelium or as a part of large “lymphoid aggregates (LAs)” (7).

**Figure 2:**
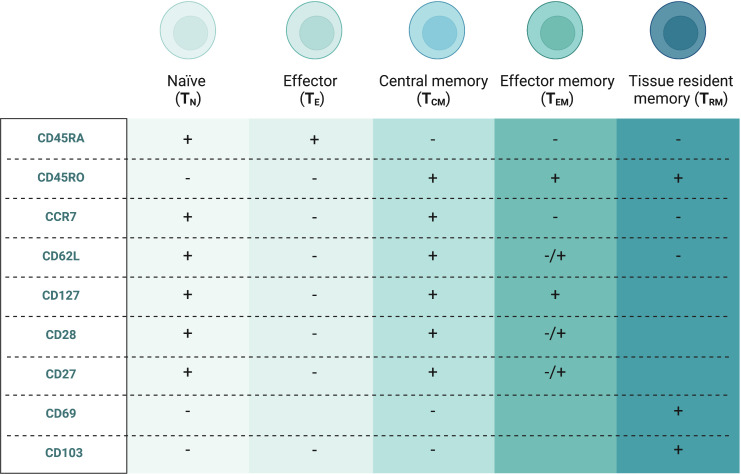
Conventional CD8+ T cell subsets in peripheral blood and endometriosis-associated tissues (10). Created with BioRender.com.

Based on their distinct thymic developmental pathways, mucosal CD8 T cells can be divided into two subsets (8, 9). The “unconventional” types such as T-cell receptor (TCR) γδ+ T cells, natural killer T (NKT) cells and mucosal-associated invariant T (MAIT) cells resemble innate immune cells and express TCRαβ/TCRγδ and typically CD8αα homodimers, while the “conventional” CD8 T cells express TCRαβ along with CD8αβ. The latter are further differentiated by multiple cell surface markers based on their function and the phases of the typical adaptive immune response as shown in **Figure 2** (10). During the initial activation and expansion, naïve CD8 T cells (T_N_) that circulate between the peripheral blood and secondary lymphoid organs recognize antigens presented via major histocompatibility complex (MHC) class I on antigen-presenting cells, start expanding and develop into effector CD8 T cells (T_E_) (11). They leave secondary lymphoid organs, secrete pro-inflammatory cytokines, such as IFN-γ and TNF-α and kill the antigen-positive cells with cytolytic molecules, such as perforins and granzymes. Most of these CD8 T cells die by apoptosis during the contraction phase. In the last phase, surviving cells develop into several forms of memory CD8 T subtypes, such as central (T_CM_), effector (T_EM_) and tissue-resident (T_RM_) memory subsets (12). T_CM_ and T_EM_ are collectively referred to as circulating memory CD8 T cells predominantly found in blood, lymph nodes and secondary lymphoid organs. Conversely, T_RM_ remain in tissue even without constant antigen stimulus and provide a rapid response to previously exposed pathogens without requiring co-stimulatory signals (13). Mucosal CD8 T cells usually acquire a tissue-resident memory phenotype, widely but not exclusively determined by CD69 and CD103 markers (14, 15).

In contrast to the mucosal tissue in the eutopic and ectopic endometrium, the peritoneal fluid mainly derives as an ovarian exudate induced by enhanced vascular permeability, but small amounts have been found in healthy men (16, 17). Although the peritoneal fluid immune milieu strongly depends on the hormonal milieu, macrophages remain the most prevalent population of peritoneal immune cells throughout the uterine cycle in women with endometriosis (55%), followed by T cells (20%) (18). Similar to the eutopic endometrium, the peritoneal fluid CD4/CD8 ratio is in favour of CD8 T cells with increased relative frequencies of this population, when compared to the peripheral blood (19).

Despite being the major T cell population in both the human endometrium and peritoneal fluid, data regarding CD8 T cells in endometriosis remain conflicting. While there have been recent publications of systematic reviews focusing on CD4+ T cells (20) and regulatory CD4+ T cells (21), there has been no systematic assessment of the data on CD8 T cells in this highly prevalent disease. Importantly, our review aims to provide the rationale for the potential role of the CD8 T population in the pathophysiology of endometriosis and associated conditions (e.g. pelvic pain, subfertility, miscarriage) as well as for its predisposition to disease recurrence.

## 2 Methods

This systematic review was registered in the international prospective database of systematic reviews - PROSPERO in January 2021 (PROSPERO ID Number: CRD42021233304) (22).

### 2.1 Database search

The methodology followed the standardised PRISMA statement guidelines for systematic reviews (23). Multiple searches were conducted from January 2021 through December 2022. Medline database (Ovid MEDLINE^®^ Epub Ahead of Print, In-Process & Other Non-Indexed Citations, Ovid MEDLINE^®^ Daily and Ovid MEDLINE^®^; 1946 to present), Embase (Ovid; 1974 to present), The Web of Science (Clarivate; 1900 to present) and CINAHL (HDAS; 1937 to present) were searched for the relevant terms from two categories: CD8 T cells and endometriosis. The following keywords were used to search text words and Medical Subject Headings (MeSH-terms): CD8, CD8+, cytotoxic T or CTL; natural killer T or NKT; mucosal-associated invariant T or MAIT; intraepithelial lymphocyte* or intra-epithelial lymphocyte*; endometriosis; endometriotic; endometrioma. Results were limited to the English language with no publication period restrictions. Additionally, relevant papers that reviewers had been aware of were also included.

### 2.2 Study selection

Duplicates were removed and all studies were screened for eligibility using the adapted population, intervention, comparator, and outcomes (PICO) framework (**Table 1**) (24). Non-peer-reviewed studies such as editorial letters, expert opinions and conference abstracts were excluded. For the first round of screening, AK and JHS independently screened the titles, abstracts, and keywords, applying eligibility criteria. If the abstract did not clearly indicate whether a study met the initial inclusion criteria, the entire article was assessed. In the second round, full records of the selected articles were retrieved and screened, and articles not complying with eligibility criteria were excluded. AK and JHS assessed papers independently through stages 1 and 2 then compared decisions and discussed discrepancies. In this instance, IG and CMB were consulted and final decisions were reached. The screening was undertaken using Rayyan QCRI (25).

**Table 1:** PICO framework for study selection.

**P**opulation	**Include: **Women between menarche and menopause with all stages of surgically confirmed endometriosis and animal models with menstrual cycle. **Exclude:** Women with adenomyosis or other gynaecological diseases, idiopathic infertility, immune diseases, undergoing immune or hormonal therapy. Women with no menstrual cycles, and animal models with oestrous cycle.
**I**ntervention	**Include: ** *In vitro* and *in vivo* studies of CD8+ T cells in endometriosis related tissues, such as but not limited to peripheral blood, peritoneal fluid, eutopic endometrium and ectopic lesions of patients and animal models with endometriosis. **Exclude:** Meta-analyses of papers (but not datasets), systematic reviews and other reviews, case reports or case series, and organizational guidelines.
**C**ontrol	**Include: ** Healthy women and animal models without endometriosis. **Exclude:** Studies with no controls, poorly defined controls, or poor controls e. g. women with adenomyosis, acute pelvic inflammatory disease, idiopathic infertility, hydro/pyo/hematosalpinx, immune diseases, undergoing immune or hormonal therapy. Women with no menstrual cycle, and animal models with oestrous cycle.
**O**utcome	**Include: ** Differences in measurable parameters (e. g. concentration, activation status, cytokine production) related to CD8+ T cells in patients and animal models with endometriosis when compared to healthy controls. Association between CD8+ T cells and disease stage, treatment response, recurrence rate after surgery, pregnancy rate, live birth rate, miscarriage rate. **Exclude:** Studies with no CD8+ T cell outcomes.

### 2.3 Data extraction

The following data were extracted from each study by AK: first author’s last name, publication year, study design, study period, sample size (patients with endometriosis and controls), participant demographics and baseline characteristics, outcomes related to CD8 T cells (e. g. concentration, activation and proliferation status, cytokine production, an association between CD8 T cells and disease stage, treatment response, recurrence rate after surgery, pregnancy rate, live birth rate, and miscarriage rate). The data were extracted into an Excel spreadsheet and were checked by JHS.

### 2.4 Quality assessment

Quality assessment of included studies was conducted independently by AK and JHS using the Newcastle-Ottawa Scale (Selection, Comparability, Exposure/Outcome) (26). Each paper was graded and defined accordingly as “good”, “fair”, or “poor” quality. Any disagreements or uncertainties were resolved with IG and CMB.

## 3 Results

### 3.1 Study selection and characteristics

**Figure 3:**
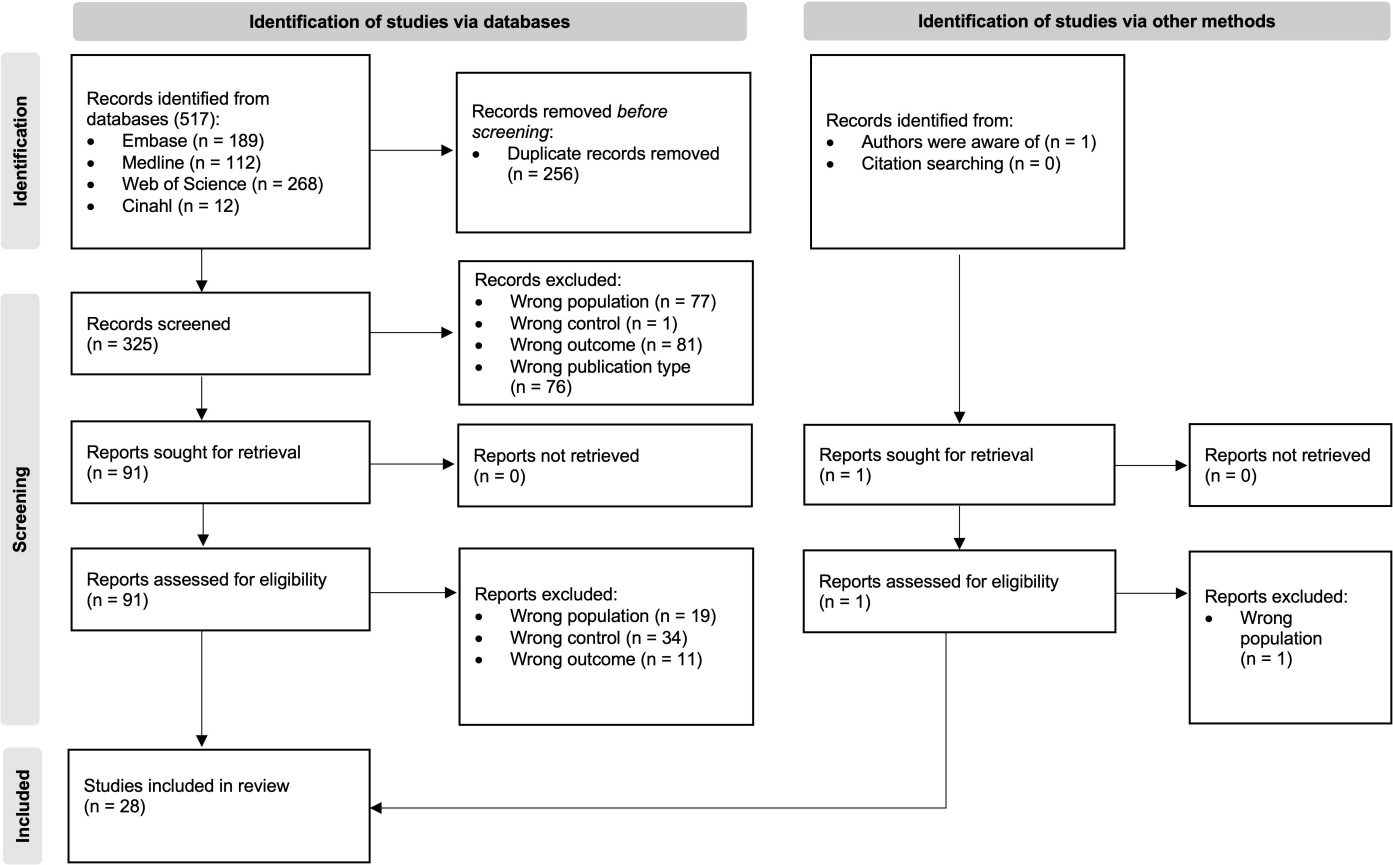
PRISMA flow diagram of study selection (23).

Initially, we identified 517 papers of which 28 met all the inclusion criteria and were included in the narrative synthesis (**Figure 3**). A detailed overview of their key characteristics is presented in **Table 2**. In summary, most studies were conducted in Asia (11), followed by Europe (8), North America (4) and South America (3), and were conducted in a university hospital setting (19).

**Table 2:** Study characteristics.

FIRST AUTHOR, YEAR	STUDY PERIOD	STUDY DESIGN				TISSUE					OBTAINING METHOD	ANALYSIS METHOD	QUALITY ASSESSMENT
		Case-control	Cohort	Transcriptome meta-analysis	Animal	Peripheral blood	Eutopic endometrium	Ectopic endometrium	Peritoneal fluid	Menstrual effluent			
Ahn, 2016	N/A	✓					✓	✓			Endometrial biopsy and laparoscopy	scRNAseq	Good
Bulmer, 1998	N/A	✓					✓	✓			Unknown for endometriosis, hysterectomy for controls	IHC	Fair
Bunis, 2022	N/A			✓			✓				Microarray data	GSEA	Good
Chen, 2017	N/A	✓				✓			✓		Venepuncture and laparoscopy	FC	Fair
Correa, 2022	2013-2015	✓				✓					Venepuncture and laparoscopy	FC	Good
D’Hooghe, 1996	N/A				✓	✓			✓		Venepuncture and laparoscopy	FC	Good
Deng, 2022	2018-2019	✓							✓		Laparoscopy	FC	Good
Gagne, 2003	1997–2001	✓				✓					Venepuncture	FC	Good
Gallinelli, 2004	1999–2001	✓							✓		Laparoscopy	FC	Poor
Gymrek, 2008	N/A	✓				✓					Venepuncture	FC	Good
Ho, 1997	1993–1996	✓				✓			✓		Venepuncture and laparoscopy	FC	Good
Hsu, 1997	1993–1994	✓				✓					Venepuncture	FC	Good
Klentzeris, 1995	N/A	✓					✓				Endometrial biopsy	IHC	Good
Li, 2019	2018–2019	✓				✓			✓		Venepuncture and laparoscopy	FC	Good
Liu, 2020	2018–2019		✓			✓					Venepuncture	FC	Fair
Ma, 2021	N/A	✓					✓	✓			Endometrial biopsy and laparoscopy	scRNAseq	Fair
Mettler, 1997	N/A	✓					✓				Endometrial biopsy	IHC	Good
Mier-Cabrera, 2011	N/A	✓				✓			✓		Venepuncture and laparoscopy	FC	Fair
Muharam, 2022	2011	✓				✓					Venepuncture	FC	Good
Opsahl, 1994	N/A	✓				✓			✓		Venepuncture and laparoscopy	FC	Fair
Poli-Neto, 2021	N/A			✓			✓				Microarray data	GSEA	Fair
Schmitz, 2021	2018-2019	✓				✓				✓	Venepuncture and menstrual cup	FC	Fair
Shih, 2022	N/A	✓								✓	Menstrual cup	scRNAseq	Fair
Sobstyl, 2020	N/A	✓				✓					Venepuncture	FC	Fair
Wu, 2019	N/A	✓					✓	✓			Venepuncture, laparoscopy and hysterectomy	IHC	Fair
Wu, 2021	2006-2013, 2016, 2018-2019	✓		✓			✓				Microarray data, N/A	GSEA, IHC	Good
Zhong, 2021	2019			✓			✓	✓			Microarray data	GSEA	Fair
Zou, 2021	N/A	✓							✓		Laparoscopy	scRNAseq	

N/A, Not available; scRNAseq, Single cell RNA sequencing; IHC, Immunohistochemistry; GSEA, Gene set enrichment analysis; FC, Flow cytometry.

### 3.2 Quality of evidence

Quality assessment was carried out for each of the included studies using the Newcastle-Ottawa Scale. Most of the papers were scored as “good” or “fair” (**Table 2**). Overall, all papers scored in the definition of cases and controls and the ascertainment of exposure. Only 2 papers reported on the non-response rate showing a potential risk of bias in the sample selection in most of the studies. One paper was graded as “low” quality due to the low score in the comparability category. One paper could not be graded as it was an animal study performed on baboons.

### 3.3 CD8+ T cells in endometriosis-associated tissues

#### 3.3.1 Eutopic endometrium

Recent bioinformatic analyses looking at several CD8 T cell subtypes found changes in TN, TCM, and TEM cell scores between the eutopic endometrium of patients and controls (**Table 3**). However, 5 out of 9 papers reported no difference in the concentration of the total CD8 T cell population between patients with endometriosis and healthy controls.

**Table 3:** CD8+ T cell outcomes in the eutopic endometrium.

AUTHORS YEAR	TOTAL (N)	ENDOMETRIOSIS (N)				CONTROLS (N)			UTERINE CYCLE PHASE (N)		ANALYZED SAMPLES OF THE TOTAL (N)		CD8+ T CELL FINDINGS IN ENDOMETRIOSIS VERSUS CONTROLS
		Stage I/II	Stage III/IV	Subfertility	Age (yrs)*	Diagnosis	Subfertility	Age (yrs)*	Proliferative	Secretory	Endometriosis	Controls	
Ahn, 2016	16	0	8	8	33.1 ( ± 7.3)	Tubal ligation	0	26 ( ± 5.8)	0	16	8	8	**NO DIFFERENCE** in the expression of CD8A gene.
Bulmer, 1998	60	N/A	N/A	N/A	N/A	Non-endometrial pathology	N/A	N/A	20	40	30	30	**NO DIFFERENCE** in neither glandular nor surface intraepithelial CD8+ cells.
Bunis, 2022	105	24	47	N/A	20–50	Tubal ligation/reanastomosis	0	20–50	47	58	71	34	**INCREASED** effector memory CD8+ T cell score across all cycle phases (FDR < 0.05, log2 fold change > 1).
Klentzeris, 1995	34	11	5	16	33.2 ( ± 1.6, 22–41)	Tubal ligation/reanastomosis	0	35.4 ( ± 2.8, 24–40)	0	34	16	18	**NO DIFFERENCE** in precipitate area for CD8+ cells.
Mettler, 1997	143	*	*	143	29.8 (24–36)	Tubal factor infertility	110	29.6 (24–35)	**	**	253	110	**NO DIFFERENCE** in CD8+ quantity per mm2 and no differences in lymphocyte aggregations.
Poli-Neto, 2021	59	16	43	N/A	N/A	Healthy	N/A	N/A	113	0	59	54	**INCREASED** effector memory CD8+ T cell score in mild and severe disease (p = 0.000; p = 0.027). **DECREASED** naive and central memory CD8+ T cell scores in in mild and severe disease (p = 0.001; p = 0.000 and p = 0.035; p = 0.782).
Wu, 2019	15	0	15	N/A	44.60 ( ± 2.57)	Non-endometrial pathology	N/A	47.00 ( ± 4.04)	30	0	15	15	**INCREASED** CD8+ quantity per mm^2^ (p < 0.01).
Wu, 2021	67	N/A	N/A	N/A	N/A	Healthy	N/A	N/A	107*	*	67	40	**INCREASED** CD8+ T cell score (CIBERSORT: p = 0.0033; MCP-counter: p = 0.00018; ImmuneCellAI: p = 7.2e-8). **DECREASED** naïve (p = 7.6e-5), central memory (p = 0.00076), and effector memory CD8+ T cells (p = 1.1e-6).
	18	N/A	N/A	9	N/A	Healthy	9	N/A	36*	*	18	18	INCREASED CD8+ T cell score in infertile women with endometriosis versus fertile women with endometriosis (p = 0.0019, AUC = 0.722).
	40	N/A	N/A	N/A	N/A	Healthy	N/A	N/A	40*	*	20	20	**INCREASED** CD8+ cell ratio (p = 0.0132, AUC = 0.727).
Zhong, 2021	180	20	62	N/A	N/A	N/A	N/A	N/A	204	0	112	68	**NO DIFFERENCE** in CD8+ T cell ratio. **NO DIFFERENCE** in CD8+ T cell ratios between mild and severe disease.

* Mixed with early secretory.

** Up to 7 patients for every day of the menstrual cycle (28–29 days).

N/A, Not available; Age (yrs)*: Mean ± SD or Median (Range).

Using gene set enrichment analysis (GSEA) on data from eutopic endometrial biopsies taken during the proliferative phase with CIBERSORT, Zhong et al. found no difference in CD8 T cell fraction between patients with severe endometriosis and controls (2). Nevertheless, in a similar analysis, investigators found increased CD8 T_EM_ cell gene enrichment scores in women with revised American Society for Reproductive Medicine (rASRM) stage I/II as well as stage III/IV disease (p = 0.000 and p = 0.027, respectively) and decreased CD8 T_N_ and T_CM_ scores (p = 0.001 and p = 0.000; p = 0.035 and p = 0.782) when compared to controls (27). Another GSEA by a different group shows an increased CD8 T cell score in endometriosis versus controls when analysed using CIBERSORT (0.1659 ± 0.0968 vs 0.1086 ± 0.0939, respectively; p = 0.0033), MCP-counter (p = 0.00018) and ImmuneCellAI (p = 7.2e-8) (28). Comparing different subsets with ImmuneCellAI, decreased CD8 T_N_ cells (p = 7.6e-5), T_CM_ (p = 0.00076), and T_EM_ cells (p = 1.1e-6) were present in the endometrium of women with endometriosis when compared with controls. In a second analysis (GSE120103), performed with MCP-counter, increased CD8 T cell scores were revealed in endometrium of infertile versus fertile women with endometriosis (p = 0.0019, AUC (area under the receiver operating characteristic curve (ROC)) = 0.722). Immunohistochemical validation of their transcriptomic data confirmed increased proportions of CD8+ cells among all leukocytes in the eutopic endometrium of endometriosis patients versus controls in both the proliferative and early secretory phases (0.2292 ± 0.0591 vs 0.1790 ± 0.0562, p = 0.0132, AUC = 0.727) (28).

Similarly, increased quantity of CD8+ cells per mm^2^ was detected in severe endometriosis versus controls, of note samples were collected in the proliferative phase (0.019 ± 0.004 vs 0.007 ± 0.002 cell/mm^2^, respectively; p < 0.01) (29). Focusing on the eutopic endometrial tissue in the secretory phase, single-cell RNA sequencing analysis did not reveal any difference in the expression of the CD8A gene between endometriosis and controls (30). Again, no difference was found in the microscopic analysis of the secretory phase tissue from infertile women with endometriosis and fertile controls (31). In samples equally distributed across the uterine cycle, no difference was found in either glandular or intraepithelial CD8+ cell count in either of the uterine cycle phases between endometriosis and controls (32), or between endometriosis and controls with laparoscopically proven tubal factor infertility resulting from prior pelvic inflammatory disease (PID) (33).

Finally, in the findings from Bunis et al., the endometrial CD8 T_EM_ signature had higher gene enrichment scores across all menstrual phases in endometriosis patients when compared to the control (34). Additionally, they were significantly increased in the mid-secretory phase when compared to the preceding phases in both disease and control (NES = 1 for unstratified samples). The activation potential of eutopic endometrial CD8 T cells was decreased in the endometriosis patients when compared to controls and genes with functions known to be relevant to phagocytosis and complement activation were stimulated in the eutopic endometrium of patients. No papers were found assessing the proliferative and cytotoxic capacities of eutopic endometrial CD8 T cells in endometriosis.

#### 3.3.2 Ectopic endometrium

All 5 studies researching ectopic endometrium reported an increased proportion of CD8 T cells in endometriotic lesions when compared to matched eutopic endometrium and/or healthy eutopic endometrium (**Table 4**). Additionally, 2 studies also investigated their activation and cytotoxic properties.

**Table 4:** CD8+ T cell outcomes in the ectopic endometrium.

FIRST AUTHOR, YEAR	TOTAL (N)	ENDOMETRIOSIS (N)				CONTROLS (N)			UTERINE CYCLE PHASE (N)		ANALYZED SAMPLES OF THE TOTAL (N)			CD8+ T CELLS FINDINGS IN ECTOPIC ENDOMETRIUM COMPARED TO EUTOPIC AND HEALTHY ENDOMETRIUM
		Stage I/II	Stage III/IV	Subfertility	Age (yrs)*	Diagnosis	Subfertility	Age (yrs)*	Proliferative	Secretory	Eutopic	Ectopic	Controls	
Ahn, 2016	16	0	8	8	33.1 ( ± 7.3)	Tubal ligation	0	26 ( ± 5.8)	0	16	8	8	8	**INCREASED** expression of CD8A gene (p < 0.05).
Bulmer, 1998	60	N/A	N/A	N/A	N/A	Non-endometrial pathology	N/A	N/A	20	40	30	30	30	**INCREASED** number in the glandular epithelium in the early secretory compared to the matched eutopic endometrium (p = 0.0002).
Ma, 2021	6	0	3	1	31.7	Non-endometrial pathology	N/A	30.4	6	0	3	3	3	**INCREASED** ratio of naive CD8+ T cells (p < 0.05). **DECREASED** ratio of effector CD8+ T cells (p < 0.05).
Wu, 2019	30	0	15	N/A	44.60 ( ± 2.57)	Non-endometrial pathology	N/A	47.00 ( ± 4.04)	30	0	15	15	15	**INCREASED** CD8+ quantity per mm^2^ when compared to control endometrium (p < 0.01). **NO DIFFERENCE** when compared to matched eutopic endometrium (p > 0.01).
Zhong, 2021	204	20	62	N/A	N/A	N/A	N/A	N/A	204	0	112	24	68	**INCREASED** CD8+ T ratio when compared to control endometrium (p = 0.033). **NO DIFFERENCE** in CD8+ T cell ratio when compared to matched eutopic endometrium.

N/A, Not available; Age (yrs)*: Mean ± SD or Median (Range).

Zhong et al. performed transcriptome meta-analysis with CIBERSORT on 24 ectopic, 82 eutopic and 68 healthy endometrial samples in the proliferative phase (GSE120103, GSE51981, GSE25628, GSE37837, GSE7846, GSE6364, GSE7305) (2). They described an increased CD8 T cell fraction among all infiltrating immune cells in the endometriosis lesions when compared to the eutopic endometrium of endometriosis patients (p = 0.085) and endometrium of controls (p = 0.033). Similarly, analyzing scRNA-seq data of proliferative phase timed samples, a significantly higher percentage of CD8 T_N_ cells was reported in endometriotic lesions when compared to matched eutopic endometrium and normal endometrium (43.48% vs 0.91% and 0.33%, respectively), and a lower proportion of T_E_ subtypes (35). In addition, the authors describe decreased cytotoxic T cell populations in lesions, however, data was not shown. Interestingly, a smaller immunohistochemistry study on proliferative phase samples confirmed increased CD8+ cell counts in ectopic endometrium when compared to the control endometrium (0.019 ± 0.002 vs 0.007 ± 0.002 per mm^2^; p < 0.01), but not in ectopic versus matched eutopic endometrium (29).

Investigating samples in the secretory phase of the menstrual cycle, genes associated with several immune cell surface markers, including CD8A, were upregulated in ectopic tissues compared with eutopic endometrium of patients as well as control endometrium (p < 0.05) (30). In the same study, they also found increased expression of immune genes for T cell co-stimulation (e. g. CD27 and CD28) as well as less specific HLA genes associated with T cell activation, such as HLA-DPA1, HLA-DPB1, HLA-DQA1, HLA-DRA, and HLA-DMA (p < 0.05). On the other hand, a different IHC study found no difference in the CD8+ cell count between the glandular epithelium of ectopic lesions and matched eutopic endometrium in the proliferative and late secretory phase but confirmed an increased count in the early secretory phase (p = 0.0002) (32).

#### 3.3.3 Peritoneal fluid

Of the 9 studies researching CD8 T cell ratios in the peritoneal fluid, 3 found no difference between endometriosis patients and controls, 1 paper noted no difference in CD8 T cell fraction among lymphocytes but increased proportions among all peritoneal cells in patients with endometriosis and 2 found increased concentrations in the disease (**Table 5**). Similarly, as shown, no consensus was reached on the activation status of the peritoneal CD8 T cells: 3 studies found no difference, 2 reported decreased and 1 increased activation in endometriosis patients.

**Table 5:** CD8+ T cell outcomes in peritoneal fluid.

FIRST AUTHOR, YEAR	TOTAL (N)	ENDOMETRIOSIS (N)				CONTROLS (N)			UTERINE CYCLE PHASE (N)		ANALYZED SAMPLES OF THE TOTAL (N)		CD8+ T CELL FINDINGS IN ENDOMETRIOSIS VERSUS CONTROLS		
		Stage I/II	Stage III/IV	Subfertility	Age (yrs)*	Diagnosis	Subfertility	Age (yrs)*	Proliferative	Secretory	Endometriosis	Controls	Cell proportion	Activation and cytotoxicity	CD4/CD8 ratio
Chen, 2017	63	14	18	N/A	29 (18–38)	Non-endometrial pathology	N/A	32 (19–44)	63	0	4	4	**NO DIFFERENCE** among CD3+ cells.		
D’Hooghe, 1996	57	20	11	N/A	N/A	Healthy	N/A	N/A	21	18	36	21	**INCREASED** in long-term spontaneous endometriosis when compared with other groups (p = 0.01).		
Deng, 2022	49	0	24	N/A	31 (± 6.8)	Non-endometrial pathology	N/A	40 ( ± 5.6)	49	0	15	18	**NO DIFFERENCE** among lymphocytes. **INCREASED** among all peritoneal fluid cells (p = 0.0032).		**NO DIFFERENCE**.
Gallinelli, 2004	63	21	22	0	33.1 ( ± 4.4)	Tubal ligation/reanastomosis	3 ± 0.6 yrs	31.7 ( ± 3.7)	80	0	0	20		**DECREASED** concentration of HLA-DR+ subsets (p < 0.02).	**DECREASED** (p < 0.05).
Ho, 1995	29	8	11	N/A	N/A	Tubal ligation	0	N/A	29	0	19	10		**NO DIFFERENCE** in HLA-DR+, CD25+, CD28+ and CD69+ subsets between mild and severe endometriosis and controls.	
Li, 2019	50	17	15	N/A	32.6 ( ± 1.10)	Non-endometrial pathology	N/A	33.3 ( ± 1.23)	50	0	29	10	**INCREASED** CD8+ MAIT cell subsets (p = 0.0454).	**INCREASED** expression of CD38 among CD8+ MAIT cells (p = 0.0071).	
Mier-Cabrera, 2011	62	32	0	32	32.7 ( ± 2.5)	Tubal ligation	0	33.8 ( ± 5.4)	0	62	32	30	**NO DIFFERENCE** among lymphocytes.	**NO DIFFERENCE** in the ratio of IFN-gamma and IL-2 expressing cells.	
Opsahl, 1994	20	8	0	8	N/A	Tubal ligation	0	N/A	11	9	8	12	**NO DIFFERENCE** among all phases.	**NO DIFFERENCE** in the ratio of HLA-DR+ subsets.	
Zou, 2021	2	0	1	N/A	36	Septate uterus	N/A	28	2	0	1	1		**DECREASED** mean gene expression of GNLY, GZMB, GZMH and GZMK (p < 0.0001).	

N/A, Not available; Age (yrs)*: Mean ± SD or Median (Range).

The proportion of CD8 T cells among total mononuclear cells, between patients with rASRM stage I/II endometriosis and fertile controls are unchanged in the proliferative and secretory phases as well as when uncontrolled for the cycle phase (36). Similarly, some authors found comparable rates of CD8 T cells among mononuclear cells in women with and without the disease in the proliferative phase (37) and others reported no difference in the percentage of CD8+ cells among all lymphocytes in the periovulatory phase between endometriosis patients and healthy controls (38). While Deng et al. identified no difference in the proportion of CD8+ cells among lymphocytes in the proliferative phase, they found increased numbers of CD8+ cells among all peritoneal fluid cells in samples from patients with endometriosis (p = 0.0032) (39).

Increased proportions of specific CD8 T cell subsets were reported in studies studying baboons and MAIT cells. In baboons, a significant increase in the proportion of peritoneal CD8 T cells was detected in the group with long-term spontaneous endometriosis, when compared to other groups with no disease, induced disease, and recent spontaneous endometriosis (74.3 ± 6% vs 67.1 ± 8.3% vs 65.8 ± 8% vs 54.8 ± 21.9%, respectively; p = 0.01) (40). In humans among MAIT cells defined as CD3+CD161+Vα7.2+ enhanced CD8+ MAIT cell subsets were found in the disease compared to control (6.52 ± 1.05 vs 2.77 ± 0.42, respectively; p = 0.0454) (41). In the same study, peritoneal fluid CD8+ MAIT cells from endometriosis patients displayed higher levels of the activation marker CD38, when compared to non-endometriosis participants (2.98 ± 0.53 vs 0.36 ± 0.09, respectively; p = 0.0071). The difference was more pronounced in the group with stages I/II than with stages III/IV when compared to controls.

On the other hand, Gallinelli et al. showed decreased proportions of CD3+CD8+HLA-DR+ cells in the endometriosis versus the control group (42.8 ± 6.8 vs 57.1 ± 7.6, respectively; p < 0.02) (42). Additionally, they found a significantly higher CD8/CD4 ratio in the disease (2.8 ± 0.5 vs 2.1 ± 0.4, respectively; p < 0.05). Nevertheless, 2 publications report no difference in activation potential between endometriosis and non-endometriosis groups (38, 43). In a study from Ho et al., they found no differences between stage I/II and III/IV endometriosis and controls in any of the identified activated phenotypes, including HLA-DR+, CD25+, CD28+, and CD69+ CD8 T cells (43).

#### 3.3.4 Menstrual effluent

Schmitz et al. investigated the variance in immune cells between menstrual effluent of patients with endometriosis and healthy controls (44). They found no statistically significant differences in either the proportion of CD8 T cells or the CD4/CD8 ratio. However, a decreased proportion of perforin-high CD8 T cells was present in the menstrual effluent of patients versus controls (4.0 ± 4.8 vs 11.2 ± 10.4, respectively; p = 0.029). In the study of Shih et al. four distinct CD8 T cell clusters were differentially enriched between patients with surgically confirmed endometriosis and community controls, although the differential markers for those four clusters have not been explored (45).

### 3.4 CD8+ T cells in peripheral blood

As presented in **Table 6**, data from 10 papers looking at the general CD8 T cell population in peripheral blood indicate no difference in ratios amongst the immune compartment between patients with endometriosis and controls, but one study on different subtypes revealed potential changes in the levels of T_CM_ and T_EM_ in the disease (46). Additionally, there seem to be no differences in their activation status between patients and controls and their proliferative capacity has not been assessed.

**Table 6:** CD8+ T cell outcomes in peripheral blood.

FIRST AUTHOR YEAR	TOTAL (N)	ENDOMETRIOSIS (N)				CONTROLS (N)			UTERINE CYCLE PHASE (N)		ANALYZED SAMPLES OF THE TOTAL(N)		CD8+ T CELL FINDINGS IN ENDOMETRIOSIS VERSUS CONTROLS	
		Stage I/II	Stage III/IV	Subfertility	Age (yrs)*	Diagnosis	Subfertility	Age (yrs)*	Proliferative	Secretory	Endometriosis	Controls	Cell proportion	Activation and cytotoxicity
Chen, 2017	63	14	18	N/A	29 (18–38)	Non-endometrial pathology	N/A	32 (19–44)	63	0	4	4	**NO DIFFERENCE** among CD3+ cells.	
Correa, 2022	73	0	47	N/A	34.3 ( ± 6.2)	Tubal ligation	N/A	34.5 ( ± 4.6)	29	44	47	26	**NO DIFFERENCE** in CD8+ iNKT cell subset.	**NO DIFFERENCE** in levels of IL-17, IL-10 or IL-6 expressing CD8+ iNKT cells.
D’Hooghe, 1996	60	20	13	N/A	N/A	Healthy	N/A	N/A	21	20	37	23	**NO DIFFERENCE** among leukocytes. **NO DIFFERENCE** among leukocytes between spontaneous and induced endometriosis.	
Gagne, 2003	306	N/A	N/A	29	31–44 (69%)	Tubal ligation/reanastomosis	0	31–44 (72%)	160	137	175	131	**NO DIFFERENCE** among leukocytes. **NO DIFFERENCE** in CD8+ cells among leukocytes.	
Gymrek, 2008	48	13	23	N/A	23–53	Non-endometrial pathology	N/A	27–51	20	28	25	11	**NO DIFFERENCE** among CD3+ cells. **NO DIFFERENCE** among CD3+ cells between different stages.	**NO DIFFERENCE** in the ratio of IFN-gamma, TNF-alpha or IL-8 expressing cells.
Ho, 1995	29	8	11	N/A	N/A	Tubal ligation	0	N/A	29	0	19	10		**NO DIFFERENCE** in HLA-DR+, CD25+, CD28+ and CD69+ subsets between mild and severe endometriosis and controls.
Hsu, 1997	52	0	26	26	26–41	Healthy	0	22–38	N/A	N/A	26	26	**NO DIFFERENCE** in CD8+CD11b- or CD8+CD11b+ level and ratio among lymphocytes.	
Li, 2019	50	17	15	N/A	32.6 ( ± 1.10)	Non-endometrial pathology	N/A	33.3 ( ± 1.23)	50	0	32	18	**NO DIFFERENCE** among MAIT population.	
Liu, 2020	101	0	33	33	30 (27–33)	Healthy	0	30 (27–32)	N/A	N/A	33	68	**INCREASED** levels of central memory subtypes (CD3+CD8+CD45RA-CCR7+; p < 0.05). **DECREASED** levels of terminally differentiated effector memory subtypes (CD3+CD8+CD28−; p = 0.006).	
Mier-Cabrera, 2011	62	32	0	32	32.7 ( ± 2.5)	Tubal ligation	0	33.8 ( ± 5.4)	0	62	32	30	**NO DIFFERENCE** among lymphocytes.	**NO DIFFERENCE** in the ratio of IFN-gamma and IL-2 expressing subsets
Muharam, 2022	14	N/A	N/A	N/A	30.57 ± 2.33	Non-endometrial pathology	N/A	33.71 ± 2.14	N/A	N/A	7	7		**NO DIFFERENCE** in CD8+CD28+ cells.
Opsahl, 1994	20	8	0	8	N/A	Tubal ligation	0	N/A	11	9	8	12	**NO DIFFERENCE** among PBMCs.	
Schmitz, 2021	23	6	6	N/A	33.7 (± 6.3)	Healthy	N/A	32.5 ( ± 7.2)	23	0	12	11	**NO DIFFERENCE** among CD3+ cells.	**NO DIFFERENCE** in the ratio of perforin high subsets.
Sobstyl, 2020	80	22	21	14	35.5 ( ± 8.0)	Healthy	N/A	36.1 ( ± 10.7)	N/A	N/A	40	40	**NO DIFFERENCE** among leukocytes. **INCREASED** CD8+TLR-2+ among CD8 T cells (p = 0.0054). **NO DIFFERENCE** in CD3+CD8+ and CD8+ TLR-2+ between different stages of endometriosis.	

N/A, Not available; scRNAseq, Single cell RNA sequencing; IHC, Immunohistochemistry; GSEA, Gene set enrichment analysis; FC, Flow cytometry; Age (yrs)*: Mean ± SD or Median (Range).

A study comparing leukocyte subsets in peripheral blood samples that were equally distributed across the menstrual cycle reported no differences in either CD8+ or CD8 T cell proportions between patients with endometriosis and healthy controls neither within the unadjusted model nor the model adjusted for age, smoking, oral contraception, parity, and history of an acute infection (CD8+: 16.4 ± 6.6 vs 16.7 ± 5.7, respectively; CD3+CD8+: 14.3 ± 5.3 vs 13.8 ± 5.7) (47). Focusing on the proliferative phase of the cycle, the median percentages of CD8 T cells among CD3+ cells and CD4/CD8 ratio in the peripheral blood between patients and healthy controls were unchanged (37) (44). Additionally, no difference was revealed in perforin-high CD8 T cells (9.0 ± 6.6 vs 12.9 ± 17.3, respectively) (44) or in the proportions of CD8 T cells expressing activation markers, such as HLA-DR, CD25, CD28 and CD69 in different disease stages when compared to controls (48). The only study investigating peripheral blood CD8 T cells solely in the secretory phase found no difference in either concentration or activation status (38).

A significant number of papers did not consider the menstrual cycle phase in their analysis. Investigators identified comparable findings with no difference in CD8 T cell proportions among leukocytes between endometriosis and control group (29.3 ± 5.5 vs 30.2 ± 4.9, respectively) and no difference in CD4/CD8 ratio (1.4 (0.73–2.7) vs 1.3 (0.93–4.5), respectively) (49). Interestingly, no difference was observed even when they compared CD8 T rates and CD4/CD8 ratio between different disease stages. Similarly, no significant changes were observed in the percentages of CD8 T cells among total mononuclear cells (36) and no variations revealed in CD8 T cell rates and CD4/CD8 ratio among CD3+ cells in endometriosis regardless of the stage (50). However, the latter group found lower expression of TNF-alpha and IFN-gamma in CD8 T cells in the endometriosis group when compared with the control group (17.4 ± 1.6 vs. 25.5 ± 2.0, respectively; p = 0.0045). Focusing on the cytotoxic activity of peripheral blood mononuclear cells, authors found no difference in CD8+CD28+ T cell ratios between endometriosis patients and controls (15.16 ± 3.26 vs 11.89 ± 2.65, respectively; p = 0.45) (51). Finally, the study on baboons discovered no differences in the CD8 T cell ratio between baboons with spontaneous endometriosis and those with the induced disease as well as between animals with the disease and healthy subjects (40).

In addition to these studies which largely compare bulk CD8 T cell ratios, some papers report on CD8 expression within other subpopulations of T cells, such as iNKT (52) and MAIT (41), or subsets of the global CD8 T repertoire such as T_CM_ and T_EM_ subtypes (46). CD8 expression on iNKT cells in combination with the regulatory cytokines IL-6, IL-17 and IL-10 were unaltered between health and endometriosis controls (52). Although Li et al. did not identify differences in the overall CD8+ MAIT population between endometriosis patients and controls (the majority of MAIT cells are CD8+), they found an increased frequency of CD8+ cells among MAIT subtypes in the peripheral blood of patients as well as controls when compared to global CD8 T cells (p = 0.003 and p = 0.0002, respectively) (41). Importantly, the only study looking into distinct CD8 T subtypes found increased levels of T_CM_ (CD3+CD8+CD45RA-CCR7+; p < 0.05) and decreased levels of terminally differentiated T_EM_ subtypes (CD3+CD8+CD28−; p = 0.006) (46).

## 4 Discussion

### 4.1 Summary

Endometriosis-associated immune alterations are complex (53). Our systematic review aimed to provide a rationale for further research into the CD8 T cells in endometriosis and associated conditions by summarizing and critically appraising the currently available data. Due to the observational study design of the included papers, this systematic review does not provide evidence for the direct involvement of the CD8 T cells in the development of endometriosis and associated disorders. Nevertheless, its findings highlight significant gaps in the literature that must be addressed in future research to determine the pathophysiological significance, non-invasive diagnostic potential and targeted treatment possibilities for this immune population. Out of 28 papers included in this systematic review, 14 could provide high-quality findings, which show that CD8 T cell levels are higher in endometriotic lesions than in the eutopic endometrium of patients as well as healthy controls, with no changes in peripheral blood levels between patients and healthy controls. However, the evidence for changes to CD8 T in peritoneal fluid and eutopic endometrium is debatable. Although older studies mostly found no difference in eutopic endometrial CD8 T cell concentrations, recent genomic analyses of distinct subtypes revealed a strong tendency of enriched CD8 T effector memory cells in the eutopic endometrium of patients with the disease. In one study investigating CD8 T cells in menstrual effluent, cytotoxicity was increased in patients while no difference was found in concentration and CD4/CD8 ratio.

### 4.2 Strengths

Numerous review articles have been published describing CD8 T cells in endometriosis (53–66). However, none of them has comprehensively investigated the literature on this immune cell population in the relevant tissues, including peripheral blood and endometriosis-associated tissues, such as eutopic endometrium, ectopic lesions, peritoneal fluid, and menstrual effluent. Our review is the first to systematically summarize adequate quality evidence of CD8 T cells in endometriosis and was based on a comprehensive search strategy and strict inclusion criteria to ensure better comparability and reliability. For example, we have excluded studies investigating participants on hormonal contraception and menopausal women since it is known that oestrogen and progesterone significantly impact the CD8 T cell milieu and might undermine the accuracy of the findings (67, 68). Additionally, we have excluded papers recruiting individuals with certain gynaecological pathologies, which could also have a significant impact on the relevant outcomes, such as adenomyosis, acute pelvic inflammatory disease, hydro/pyo/hematosalpinx, idiopathic infertility, and immune diseases (69–72).

### 4.3 Limitations

Despite the strict inclusion criteria, one of the weaknesses of this systematic review is that it is based on observational studies with small sample sizes which did not consistently account for confounders, so bias cannot be ruled out. While it is widely accepted that the menstrual cycle phase influences CD8 T cell counts and activation status in endometriosis-associated tissue, one of the included studies also found a clear correlation between the levels of several blood leukocytes and age, parity, previous use of oral contraceptives, smoking, and recent history of acute infection, but no other studies accounted for this (5, 47). With surgery being the gold diagnostic standard, it was expected that this review would suffer from the heterogeneity of the control population, with 8 studies having recruited control participants with various gynaecological conditions, such as uterine fibroids, ovarian cysts, and tubal factor infertility, and 4 of them including “healthy” controls without having undergone surgery to rule out potential pathological conditions. Additionally, some participants might have undergone several diagnostic and therapeutic surgeries before being enrolled in the study, which might have impacted the outcomes as significant immune changes are associated with the postoperative period (73).

### 4.4 Potential roles of CD8+ T cells in endometriosis

Imaging techniques have recently been recognized as a reliable diagnostic alternative to invasive procedures but they still have significant limitations for the detection of early disease (74). Therefore, the development of novel non-invasive biomarkers is urgently needed to provide alternatives to surgery for diagnosis, and to reduce the prolonged time to diagnosis that is widely recognised for this condition (75). Furthermore, biomarkers are a dynamic and effective tool to understand the range of the disease and provide a means for a consistent disease and risk factor assessment, which could help us learn more about the underlying aetiology of the disease (76). Regarding the pathophysiology, none of the studies included in this review were designed to elucidate whether an altered CD8 T population could be the cause or the consequence of endometriosis. The closest is the study by D’Hooghe et al. in which they investigated peripheral blood and peritoneal fluid of baboons with spontaneous and induced disease compared to healthy controls (40). While no difference was found in blood, an increased CD8 T cell ratio was found in peritoneal fluid of long-term spontaneous endometriosis when compared to animals with the recent spontaneous disease, induced disease and healthy controls, which could point out to the altered immune environment being a consequence rather than a cause of the disease. No conclusions can be drawn from human studies, which did not find any differences in the CD8 T cells between different disease stages and healthy controls.

#### 4.4.1 Local findings

The eutopic endometrium is an important mucosal barrier and the location of embryo implantation. Not only is immune dysregulation in eutopic endometrium associated with subfertility it may also play a role in lesion establishment (77, 78). Therefore, along with other immune cell populations, CD8 T cells are an important consideration in endometriosis. Although the data regarding eutopic endometrial CD8 T cells in endometriosis altogether are controversial, our systematic review gives an overview not only of the whole CD8 T cell population, but of alterations in other CD8 T cell subtypes in the disease. CD8 TN and TCM cell gene enrichment scores were found to be decreased while TEM cells seem to be increased (27, 28, 34). There is increasing evidence demonstrating an association between endometriosis and autoimmunity and CD8 T_EM_ cells are likely an important player in the pathogenesis of autoimmune diseases due to their effectiveness and durability (72). Nevertheless, evidence is not uniform in other autoimmune disease where for example, CD8 T_EM_ cells are increased in the cerebrospinal fluid of patients with multiple sclerosis. However the opposite was found in the gut of patients with inflammatory bowel disease (79, 80). More research is certainly needed to ascertain their role in the pathogenesis of endometriosis (81).

T_RM_ are thought to be the most prevalent CD8 T subpopulation in mucosal tissues but our understanding of this subtype in the human endometrium is poor (12). T_RM_ in the gut are widely researched and there is strong evidence they are implicated in the pathogenesis of inflammatory bowel disease (IBD) and other intestinal immunopathologies such as coeliac disease (13, 82). For example, authors investigating T_RM_ in IBD show considerably lower levels of CD103+ T_RM_ in patients with flares versus patients with endoscopic remission and healthy controls, which suggests their contribution to tissue homeostasis and immune regulation (83). Higher levels of CD69+CD103+ T_RM_ were associated with active disease in patients with vitiligo, and importantly, these cells can attract cytotoxic effector CD8 T cells from the blood, which is critical for disease persistence (84). In other gynaecological pathologies, authors revealed a modified eutopic endometrial CD8 T_RM_ phenotype in recurrent miscarriage with considerably lower expression of tissue residency marker CD69 (85).

In adenomyosis, CD8 T cell subsets are increased not only in the eutopic, but also in the ectopic endometrium of patients compared to healthy controls (86). Similar seems to be true for endometriosis, although there are discrepancies in the results between the lesions and matched eutopic endometrium. However, we emphasize that studies reporting no difference in CD8 T cells between eutopic and ectopic endometrium did not investigate specific phenotypes (2, 29). Although the potential roles of CD8 T cells in lesions are not fully defined, some parallels may be drawn from cancer research, where CD8 T cells are regarded as the most potent effectors in the anti-cancer immune response and form the basis of some current cancer immunotherapies (87). For example, increased intraepithelial CD8 T cell count in the tumour microenvironment is associated with prolonged survival in colorectal, ovarian and endometrial cancer (88–90) and the intraepithelial subpopulation of CD8 T cells was found to be increased in ectopic lesions in the early secretory phase when compared to matched eutopic and healthy endometrium (32). However, no studies were conducted comparing intraepithelial CD8 T cells between lesions of patients with different endometriosis stages and between those who benefited from the surgery or relapsed, which would give us valuable insights into the prognostic role of this population.

It has been suggested that endometriotic lesions ought to be investigated along with peritoneal fluid, since this dynamic environment strongly influences their pathophysiology (91). Nevertheless, to date, studies have examined lesions and peritoneal fluid in isolation. Similar to eutopic endometrium, data on peritoneal CD8 T cells are contradictory and no definitive conclusions can be made about the role of the CD8 T cell population in this regard. On the other hand, macrophages are a well-researched population in this setting as they are the most abundant peritoneal immune cell population, representing around 50% of peritoneal cavity leukocytes (92). They are required for lesion growth, development, vascularization, and innervation, and are even associated with pain symptoms. In endometriosis, an increased ratio of altered peritoneal macrophages has been indicated, and since they are involved in antigen presentation to CD8 T cells, this points to potentially related dysfunctions within the CD8 T cell compartment. Similarly, changes were found in regulatory T cells (Tregs) in endometriosis, with findings showing an increased number of activated Tregs in the peritoneal fluid of patients with endometriosis (93). Like macrophages, Tregs are involved in CD8 T cell activation and play an important role in the generation of high-avidity initial responses and effective memory (94).

One aim of this review was to gain a better understanding of the activation characteristics of the CD8 T cells in endometriosis. Unfortunately, no functional analyses have been performed to date on either eutopic or ectopic endometrial CD8 T cells, despite clear evidence of endometriosis-related dysfunctions in key regulators of CD8 T function, such as programmed cell death protein 1 (PD-1), programmed death-ligand 1 (PD-L1) and CD4+ T regulatory cells (29, 53, 95). Functional analyses of CD8 T cells in peritoneal fluid demonstrate inconsistent findings, where data mostly report no difference or decreased activation potential of peritoneal CD8 T cells with only one study reporting an increase in activated CD8+ MAIT cells. There is some evidence of upregulated activation marker CD69 expression on peritoneal fluid T cells in endometriosis patients, although it is important to emphasize that the investigated cohort had been taking hormonal treatment hence this study was not included in our systematic review (19).

Studies on immune cells in the female genital tract are challenging given the invasive nature of collecting mucosal and peritoneal samples (96). As previously mentioned, most research depends on biopsies and hysterectomies from women with underlying pathologies and samples from healthy women are limited. Menstrual effluent contains cells of endometrial origin and could be a valuable non-invasive source of endometrial immune cells, but has so far been overlooked in endometriosis research (44). Authors found decreased expression of perforin in CD8 T cells of patients when compared with controls and argued that perforin-mediated cytotoxicity may play a crucial role in the establishment of the lesions, drawing from research on CD8 T cells in susceptibility and elimination of cancer cells. Importantly, this could provide some answers to why only a few women develop endometriosis while retrograde menstruation occurs in most females of reproductive age (97). However, further investigation of menstrual effluent is required to understand the processes responsible for endometrial cell attachment and development outside of the endometrium, especially given the key differences in immune milieu between eutopic and ectopic endometrium and peritoneal fluid which we have confirmed in our review.

#### 4.4.2 Systemic findings

The ease of sampling makes peripheral blood one of the most studied tissues in endometriosis leading to valuable insights into the systemic nature of endometriosis and its association with autoimmune diseases, including abnormalities in the T cell population (72, 93, 98). Peripheral blood immune cells are influenced by sex hormones, with higher CD8 T cell numbers in males, but greater activation, proliferation and cytotoxic capacity in females (99). Although longitudinal studies investigating CD8 T in the peripheral blood at different menstrual phases are limited, modifications in Treg cells (100) and Th1/Th2 ratios (101) indicate that it is important to consider the menstrual cycle when investigating CD8 T population in the peripheral blood. However, this was not the case in some of the included studies. While most of our data does not suggest discrepancies in the peripheral CD8 T cells, it is important to highlight that most papers did not describe CD8 T cell subpopulations. They were shown to be significantly changed in patients in one of the recent analyses with increased central memory and decreased effector memory subtypes in the disease (46). Interestingly, several authors confirmed a decreased frequency of peripheral effector memory CD8 T cells in patients with multiple sclerosis at the onset of the disease and throughout its clinical course, which underlines its importance in the initiation of the disease rather than being its consequence (102). Additionally, there is evidence of changes in innate immune cells in the peripheral blood of patients with endometriosis, such as monocytes, which are important for antigen presentation and CD8 T cell activation (103). Reduced numbers of classical and intermediate monocytes were found in the peripheral blood of endometriosis patients, while the levels of plasmacytoid dendritic cells and non-classical monocytes were increased. Nevertheless, to better understand the systemic role of CD8 T cells in endometriosis, detailed phenotypic studies are needed, which will address specific subtypes and their activation characteristics found to be changed in the peripheral blood of other immune diseases (84).

#### 4.4.3 Endometriosis-associated subfertility

Although the pathophysiology of endometriosis-associated subfertility remains uncertain, several findings support altered endometrial immune receptivity as one of the possible causes (77, 104). The exact nature of CD8 T in eutopic endometrium is poorly understood, but the suggested hormonal influence on LA formation and CD8 T cell cytotoxicity underline the importance of optimal regulation during implantation and pregnancy (5, 68). As noted in our review, authors found significantly increased CD8 T cell scores in infertile women with endometriosis when compared to fertile women as well as in infertile versus fertile women with or without endometriosis (28). Despite the limited sample size of this GSEA analysis, it reveals possible changes in CD8 T cells in patients with endometriosis-associated infertility and urges for more investigation into the phenotypic characterisation of the population in this related disorder. It is likely that different CD8 T phenotypes could contribute to subfertility and pregnancy complications as their findings correspond to the results of several authors, who confirmed altered endometrial CD8 T characteristics in endometrial biopsies obtained during the window of implantation from patients with recurrent pregnancy loss, pre-eclampsia, and preterm labour (85, 105–107).

### 4.5 Future research

By combining cutting-edge technologies, such as single-cell RNA sequencing, mass cytometry and spectral flow cytometry with established high-resolution, high-plex and multi-omic spatial biology solutions, such as spatial transcriptomics and multiplex immunostaining, we could spatially verify immune phenotypes, their functional capacities, and cell-cell interactions to ascertain the physiological and pathophysiological function of endometrial CD8 T cells. Importantly, structures similar to LA and intraepithelial lymphocytes are known to play a role in intestinal inflammatory disorders therefore a deeper understanding of endometrial LA and intraepithelial CD8 T subsets may finally unravel their involvement in endometrial pathologies and infertility. For example, Tan et al. undertook image mass cytometry in addition to single-cell transcriptomic characterization of ectopic and eutopic endometrium, providing a cell atlas of the endometriosis microenvironment (108). This is a promising direction for future research and the analysis of a larger cohort, including those not taking hormonal contraceptives, will enable us to draw conclusions on the disease pathophysiology and contribute critical information for future diagnostics and therapies. Importantly, further research should also provide more detailed insights into menstrual effluent, as well as functional and cytotoxic properties of the population in relevant tissues with consistent considerations for the phase of the uterine cycle and parity.

### 4.6 Conclusions and clinical implications

Understanding of CD8 T cells in endometriosis is limited, with significant discrepancies in current data in endometriosis-associated tissues. We have identified relevant gaps in the literature, such as deficient phenotypic and functional analyses in all relevant tissues. In the future, a detailed pathophysiological characterization could lead to the discovery of non-invasive diagnostic biomarkers as well as successful drug repurposing and the development of targeted treatments to alter the immunological niche for better lesion clearing as well as endometrial homeostasis, vital for pregnancy success.

## Data availability statement

The original contributions presented in the study are included in the article/**Supplementary Material**. Further inquiries can be directed to the corresponding author.

## Author contributions

AK contributed to all phases of the study, including its design, data collection, screening, analysis, and interpretation, as well as the paper’s drafting. Two rounds of screening, data extraction and grading were simultaneously handled by JHS, who also participated in data interpretation and synthesis. IG and CMB assisted with the screening process and interpretation of the results. All authors contributed to the article and approved the submitted version.
