# Supplementary material for: The role of CD8+ T cells in endometriosis: a systematic review
Source: Front Immunol. 2023 Jul 11;14:1225639. doi: 10.3389/fimmu.2023.1225639 (PMC10366819; doi:10.3389/fimmu.2023.1225639)
Supplement: Supplementary file 2 [file DataSheet_2.pdf]

1st round screening criteria: Title, abstract and keywords

<table>
<tr><td>Population</td><td>Include
Women between menarche and menopause with all stages of endometriosis and animal models with oestrous cycle.
Population search terms: endometriosis, endometriotic, endometrioma, ectopic endometrium

Exclude
Wrong population: Articles that do not include our population search terms</td></tr>
<tr><td>Intervention</td><td>Include
In vitro and in vivo studies of CD8+ T cells in endometriosis related tissues, such as but not limited to peripheral blood, peritoneal fluid, eutopic endometrium and ectopic lesions of patients and animal models with endometriosis.

Exclude
Wrong publication type:
Meta-analysis
Systematic reviews and other reviews
Case reports or case series
Organizational guidelines
Editorial letters
Expert opinions
Conference papers</td></tr>
<tr><td>Outcome</td><td>Include
Differences in measurable parameters (e. g. concentration, activation status, cytokine production) related to CD8+ T cells in patients and animal models with endometriosis when compared to healthy controls. Association between CD8+ T cells and disease stage, treatment response, recurrence rate after surgery, pregnancy rate, live birth rate, miscarriage rate.
Outcome search terms: CD8 or CD8+ or cytotoxic T or CTL, natural killer T or NKT, mucosal-associated invariant T or MAIT, intraepithelial lymphocyte* or intra-epithelial lymphocyte*, tissue resident memory T or Trm

Exclude
Wrong outcome: Articles that do not include our outcome search terms</td></tr>
</table>

1st round screening algorithm

Screening titles, abstracts and keywords for search terms

	JS Included	JS Excluded	JS Maybe
**AK Included**	Included.	Full text screening for search terms by AK. Discussion if unclear.	Full text screening for search terms by AK. Discussion if unclear.
**AK Excluded**	Included, unless excluded by AK as conference abstracts.	Excluded.	Full text screening for search terms by AK. Discussion if unclear.
**AK Maybe**	Full text screening for search terms by AK. Discussion if unclear.	Excluded.	Full text screening for search terms by AK. Discussion if unclear.

2nd round screening criteria: Full text

Population

Include
Women between menarche and menopause with all stages of endometriosis and animal models with oestrous cycle.
Exclude
Wrong population:
1. Does not meet inclusion criteria
2. Ineligible population characteristics:
    - No menstrual or oestrous cycle
    - Hormonal treatment
    - Adenomyosis or other gynaecological disease
    - Idiopathic infertility
    - Immune disease or immune therapy
    - Thyroid disease

Intervention

Include
In vitro and *in vivo* studies of CD8+ T cells in endometriosis related tissues, such as but not limited to peripheral blood, peritoneal fluid, eutopic endometrium and ectopic lesions of patients and animal models with endometriosis.
Exclude
Wrong intervention:
1. Does not meet inclusion criteria
2. Wrong publication type
    - Meta-analysis
    - Systematic reviews and other reviews
    - Organizational guidelines
    - Editorial letters
    - Expert opinions
    - Conference papers
3. Wrong study design
    - Case reports
    - Case series

Control

Include: Healthy women and animal models.
Exclude
Wrong control group:
1. No control group
2. Ineligible control group characteristics:
    - No menstrual or oestrous cycle
    - Hormonal treatment
    - Adenomyosis or other gynaecological disease
    - Idiopathic infertility
    - Immune disease or immune therapy
    - Thyroid disease

Outcome

Include
Differences in measurable parameters (e. g. concentration, activation status, cytokine production) related to CD8+ T cells in patients and animal models with endometriosis when compared to healthy controls. Association between CD8+ T cells and disease stage, treatment response, recurrence rate after surgery, pregnancy rate, live birth rate, miscarriage rate.
Exclude
Wrong outcome:
Does not meet inclusion criteria
Other

2nd round screening algorithm

Screening full text papers against established PICO criteria

	**JS Included**	**JS Excluded**	**JS Maybe**
**AK Included**	Included.	Discussion with final agreement.	Discussion with final agreement.
**AK Excluded**	Discussion with final agreement.	Excluded.	Discussion with final agreement.
**AK Maybe**	Discussion with final agreement.	Discussion with final agreement.	Discussion with final agreement.
