# Supplementary material for: The role of CD8+ T cells in endometriosis: a systematic review
Source: Front Immunol. 2023 Jul 11;14:1225639. doi: 10.3389/fimmu.2023.1225639 (PMC10366819; doi:10.3389/fimmu.2023.1225639)
Supplement: Supplementary file 3 [file DataSheet_3.pdf]

NEWCASTLE - OTTAWA QUALITY ASSESSMENT SCALE
CASE CONTROL STUDIES

Note: A study can be awarded a maximum of one star for each numbered item within the Selection and Exposure categories. A maximum of two stars can be given for Comparability.

Selection

1) Is the case definition adequate?
   a) yes, with independent validation (e.g. >1 person/record/time/process to extract information, or reference to primary record source such as x-rays or medical/hospital records)*
   b) yes, e.g. record linkage (e.g. ICD codes in database) or based on self-reports
   c) no description

2) Representativeness of the cases
   a) consecutive or obviously representative series of cases (All eligible cases with outcome of interest over a defined period of time, all cases in a defined catchment area, all cases in a defined hospital or clinic, group of hospitals, health maintenance organization, or an appropriate sample of those cases (e.g. random sample))*
   b) potential for selection biases or not stated

3) Selection of Controls
   a) community controls (i.e. same community as cases and would be cases if had outcome)*
   b) hospital controls (within same community as cases (i.e. not another city) but derived from a hospitalised population)
   c) no description

4) Definition of Controls
   a) no history of disease (endpoint)*
   b) no description of source

Comparability

1) Comparability of cases and controls on the basis of the design or analysis
   a) study controls for uterine cycle*
   b) study controls for any additional factor* (age, fertility)

Either cases and controls must be matched in the design and/or confounders must be adjusted for in the analysis. Statements of no differences between groups or that differences were not statistically significant are not sufficient for establishing comparability. Note: If the odds ratio for the exposure of interest is adjusted for the confounders listed, then the groups will be considered to be comparable on each variable used in the adjustment.
There may be multiple ratings for this item for different categories of exposure (e.g. ever vs. never, current vs. previous or never)

Notes:
UTERINE CYCLE: If they’re all from the same cycle or analysed separately.
FERTILITY: If controls are proven to be fertile (e. g. tubal ligation).

Exposure

1) Ascertainment of exposure

   a) secure record (eg surgical records) or structured interview where blind to case/control status*

   c) interview not blinded to case/control status

   d) written self-report or medical record only

   e) no description

2) Same method of ascertainment for cases and controls

   a) yes*

   b) no

3) Non-Response rate

   a) same rate for both groups*

   b) non respondents described

   c) rate different and no designation
